# Gemstone spectral imaging: determination of CT to ED conversion curves for radiotherapy treatment planning

**DOI:** 10.1120/jacmp.v14i5.4335

**Published:** 2013-09-06

**Authors:** Masashi Yagi, Takashi Ueguchi, Masahiko Koizumi, Toshiyuki Ogata, Sachiko Yamada, Yutaka Takahashi, Iori Sumida, Yuichi Akino, Koji Konishi, Fumiaki Isohashi, Noriyuki Tomiyama, Yasuo Yoshioka, Kazuhiko Ogawa

**Affiliations:** ^1^ Department of Radiation Oncology Osaka University Graduate School of Medicine Osaka; ^2^ Department of Radiology Osaka University Hospital Osaka; ^3^ Division of Medical Physics Oncology Center Osaka University Hospital Osaka; ^4^ Department of Oral and Maxillofacial Radiology Osaka University Graduate School of Dentistry Osaka; ^5^ Department of Diagnostic and Interventional Radiology Osaka University Graduate School of Medicine Osaka Japan

**Keywords:** dual‐energy CT, Gemstone spectral imaging, monochromatic image, radiotherapy treatment planning, CT to ED conversion curve

## Abstract

The monochromatic images acquired by Gemstone spectral imaging (GSI) mode on the GE CT750 HD theoretically determines the computed tomography (CT) number more accurately than that of conventional scanner. Using the former, the CT number is calculated from (synthesized) monoenergetic X‐ray data. We reasoned that the monochromatic image might be applied to radiotherapy treatment planning (RTP) to calculate dose distribution more accurately. Our goal here was to provide CT to electron density (ED) conversion curves with monochromatic images for RTP. Therefore, we assessed the reproducibility of CT numbers, an important factor on quality assurance, over short and long time periods for different substances at varying energy. CT number difference between measured and theoretical value was investigated. The scanner provided sufficient reproducibility of CT numbers for dose calculation over short and long time periods. The CT numbers of monochromatic images produced by this scanner had reasonable values for dose calculation. The CT to ED conversion curve becomes linear with respect to the relationship between CT numbers and EDs as the energy increases. We conclude that monochromatic imaging from a fast switching system can be applied for the dose calculation, keeping Hounsfield units (HU) stability.

PACS numbers: 87.55.‐x, 87.55.ne, 87.57.N‐, 87.59.bd

## I. INTRODUCTION

The application of computed tomography (CT) in radiotherapy is growing and plays an important role for radiotherapy. In radiotherapy, the information acquired by the CT scanner is mainly used to identify targets and organs at risk (OARs) and determine appropriate dosing.^(1)^

Recently, dual‐energy CT has been commercially available. The dual‐energy CT concept was suggested by Hounsfield in 1973.^(2)^ Technological limits such as low rotation speed made it initially difficult to use the scanner clinically. However, subsequent rapid advances in CT technology have resulted in the ubiquitous clinical presence of dual‐energy scanners. Dual‐energy CT can provide more valuable information such as effective atomic number, electron density, and mass density^(^ ^3^ ^,^ ^4^ ^,^ ^5^ ^)^ than that acquired by conventional scanners. Furthermore, the monochromatic X‐ray image acquired by the dual‐energy scanner theoretically yields more accurate data than that of the conventional scanner because the theory (details in the paragraph below) predicts the elimination of spectral beam hardening artifacts.^(^ ^5^ ^,^ ^6^ ^,^ ^7^ ^)^ We reasoned that the monochromatic image could make more accurate dose distribution calculations when applied to radiotherapy treatment planning (RTP). Therefore, we set out to validate this technique.

There are three types of dual‐energy CT acquisition systems commercially available: Toshiba (Toshiba Corporation, Tokyo, Japan) with one tube and two rotations, Siemens^(8)^ (Siemens Medical Solutions, Malvern, PA, USA) with two tubes and one rotation, and GE^(9)^ (GE Healthcare, Waukesha, WI, USA) with one tube one rotation called a fast switching system. The similar system to that from GE is described in several papers.^(^ ^7^ ^,^ ^10^ ^,^ ^11^ ^)^ One of the most important requirements for the success of dual‐energy CT scanning is that there should be minimal time delay between the two acquisitions of the two single energy projection/images. We utilize a Discovery CT750 HD (GE Healthcare). In the GE system, fast rotation speed (0.5–1 s), use of X‐ray focal spot deflection, fast voltage switching speed between 80 and 140 kVp in less than 0.5 ms, and a newly developed cerium activated garnet rare‐earth composite scintillator detector with 100 times faster response than a typical gadolinium oxysulfide CT detector allow us to perform successful dual‐energy CT acquisition.

The basic theory of this algorithm was first reported by Alvarez and Macovski^(6)^ and studied extensively by others.^(^ ^12^ ^,^ ^13^ ^)^ The system uses a dual‐energy pre‐reconstruction algorithm for creating synthesized monochromatic CT image from the material density images. The following is an explanation of the method.^(^ ^7^ ^,^ ^9^ ^)^ The basic assumption underlying this algorithm is that over the diagnostic X‐ray energy range, the explicitly energy‐dependent linear attenuation coefficient of all materials can be expressed with sufficient accuracy as a linear combination of photoelectric and Compton coefficients.^(6)^ In direct consequence, the linear attenuation coefficient in each voxel of CT image at energy, E, is given by:

(1) $$\mu^{L}(E)=d_{\alpha }\cdot \mu^{M}{\left(E\right)}_{\alpha }+d_{\beta }\cdot \mu^{M}{\left(E\right)}_{\beta }$$

where μ(*E*) is linear attenuation coefficient in each voxel at X‐ray energy *E* (in kVp), and $d_{\alpha }$ and $d_{\beta }$ are the dual‐energy CT determined densities or concentrations of basis materials aand β at the voxel location, respectively. Thus, the information of two materials is needed to calculate the linear attenuation coefficient. It should be sufficiently different in their atomic number Z and in their photoelectric and Compton attenuation characteristics to distinguish the two materials. $\mu^{M}{\left(E\right)}_{\alpha }$ and $\mu^{M}{\left(E\right)}_{\beta }$ are the mass attenuation coefficients of material α and β In CT, the line integral over the linear attenuation coefficient $\int \mu^{L}(r,E)ds$ is determined for each focus position and detector element, respectively. This integral can be expressed accordingly as:

(2) $$\int \mu^{L}(r,E)ds=\delta_{\alpha }\cdot \mu^{M}{\left(E\right)}_{\alpha }+\delta_{\beta }\cdot \mu^{M}{\left(E\right)}_{\beta }$$

where

(3) $$\delta_{i}=\int d_{i}(r)(ds)$$

$\delta_{i}$ is the area density in $g/{\text{cm}}^{2}$ and $d_{i}(r)$ is the local density in $g/{\text{cm}}^{3}$ of the basis material *i*.

The equivalent area densities, $\delta_{\alpha }$ and $\delta_{\beta }$ are determined for each ray path in projections. This problem is solved by measuring the attenuation with two different energies (spectra). Because the X‐ray attenuates according to Beer‐Lambert law (i.e., exponential attenuation law):

(4) $$I=I_{0}(E)\cdot \text{exp}\left[-\int \mu^{L}(r,E)ds\right]$$

where *I* and $I_{0}$ are the attenuated and primary intensities, two nonlinear equations for each path are derived:

(5) $$I_{h}=I_{0h}(E)\cdot \text{exp}\left[-\delta_{\alpha }\cdot \mu^{M}{\left(E\right)}_{\alpha }-\delta_{\beta }\cdot \mu^{M}{\left(E\right)}_{\beta }\right]dE$$

(6) $$I_{l}=I_{0l}(E)\cdot \text{exp}\left[-\delta_{\alpha }\cdot \mu^{M}{\left(E\right)}_{\alpha }-\delta_{\beta }\cdot \mu^{M}{\left(E\right)}_{\beta }\right]dE$$

The subscripts *h* and *l* refer to the high‐ and low‐kVp energy. Eqs. (5) and (6) can be solved for the equivalent area densities, $\delta_{\alpha }$ and $\delta_{\beta }$.

Since mass attenuation coefficient would have been measured with a mono‐energetic X‐ray source,^(14)^ once the equivalent area densities are determined, the projection data can be calculated. This is done by multiplying the known area density values by the mass attenuation coefficients of the respective basis materials for an arbitrary mono‐energy $E_{0}$ (in keV):

(7) $$\int \mu^{L}(r,E_{0})ds=\delta_{\alpha }\cdot \mu^{M}{\left(E_{0}\right)}_{\alpha }+\delta_{\beta }\cdot \mu^{M}{\left(E_{0}\right)}_{\beta }$$

where $\mu^{M}{\left(E_{0}\right)}_{i}$ are taken from Storm and Israel.^(14)^ The projection data are then subjected to the standard reconstruction process yielding CT images in Hounsfield units (HU).^(15)^

In conventional CT, the HU or CT number (CT#) is computed as:

(8) $$CT\#(E)=\frac{\mu^{L}(E)-\mu^{L}{\left(E\right)}_{w}}{\mu^{L}{\left(E\right)}_{w}}.1000$$

where $\mu^{L}{\left(E\right)}_{w}$ is the linear attenuation of pure water at a given energy E. In dual‐energy CT, to calculate *CT#* at $E_{0}$, the equation is transformed using the linear mass relationship $\mu^{L}\;=\;\rho \mu^{M}$ and substituted Eq. (1) into Eq. (8):

(9) $$CT\#(E_{0})=\frac{d_{\alpha }\cdot \mu^{M}{\left(E_{0}\right)}_{\alpha }+d_{\beta }\cdot \mu^{M}{\left(E_{0}\right)}_{\beta }-\rho_{w}\cdot \mu^{M}{\left(E_{0}\right)}_{w}}{\rho_{w}\cdot \mu^{M}{\left(E_{0}\right)}_{w}}\cdot 1000$$

where $\rho_{w}$ and $\mu^{M}{\left(E_{0}\right)}_{w}$ are the pure water mass density and mass attenuation coefficient of pure water at energy $E_{0}$, respectively. This monochromatic image synthesis workflow is implemented in Gemstone spectral imaging (GSI) mode while standard polychromatic images were obtained using the regular operation mode.

The goal of this study is to provide CT to ED (electron density) conversion curves derived from dual‐energy CT monochromatic images for RTP. The CT number accuracy, as well as the reproducibility of CT numbers (an important factor on quality assurance), was also investigated.

## II. MATERIALS AND METHODS

### A. Phantom

**Figure 1:**
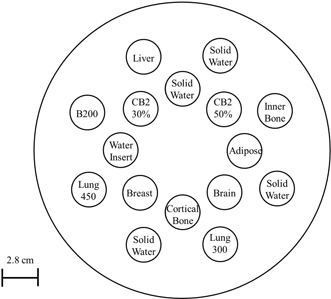
Phantom rod alignment. The high‐density materials were positioned to minimize artifacts.

A tissue characterization phantom (Gammex RMI 467, Gammex RMI, Middleton, WI, USA) with 33 cm in diameter and 5 cm in height was used. The rods' compositions mimicked those of human body organs with known electron densities relative to water, ranging from low (e.g., air) to high (e.g., bone). The phantom was composed mainly of solid water. In radiotherapy, this phantom is commonly used to establish EDs of various tissues and their corresponding CT numbers (in Hounsfield units, HU) for accurate corrections for tissue heterogeneity. Table 1 summarizes the physical characteristics of the rods (provided by the manufacturer to compensate slight differences among products). Correctly aligning rods is quite important because high‐density rods cause artifacts that affect the accuracy of the CT numbers. Therefore, rods were inserted into the phantom according to the manufacture's recommendations (Tissue Characterization Phantom Model 467 User's Guide).^(16)^ Figure 1 shows the distribution of the rods used in this study.

**Table 1:** Physical characteristics of rods. The data are listed in ascending order by electron density

*Rod Material*	Electron Density Relative to Water	Physical Density $\left(g/{\text{cm}}^{3}\right)$
LN‐300 Lung	0.284	0.290
LN‐450 Lung	0.445	0.460
AP6 Adipose	0.924	0.941
BR‐12 Breast	0.957	0.980
CT Solid Water	0.988	1.017
Water Insert	1.000	1.000
BRN‐SR2 Brain	1.049	1.053
LV1 Liver	1.062	1.094
IB Inner Bone	1.097	1.144
B200 Bone Mineral	1.096	1.143
$\text{CB}2‐30\%\;{\text{CaCO}}_{3}$	1.279	1.334
$\text{CB}2‐50\%\;{\text{CaCO}}_{3}$	1.470	1.560
SB3 Cortical Bone	1.696	1.824

### B. Measurements

The phantom was carefully placed at the isocenter of the gantry using well‐coordinated laser pointer system to ensure that the central axis and transverse plane of the phantom were precisely on the longitudinal axis and imaging plane, respectively. Since helical scanning was typically used in the CT simulation for RTP, all measurements were acquired by helical scanning in either Regular or GSI mode. Table 2 summarizes the scan parameters. The standard 120 kVp image was reconstructed using Regular mode with the parameters listed in Table 3. The monochromatic images at 60 keV (relatively lower setting), 77 keV, 100 keV, and 140 keV (the highest setting) were reconstructed using GSI mode with the parameters listed in Table 3, where 77 keV is approximately equal to the effective energy of a 120 kVp polychromatic X‐ray beam, according to the scanner specifications.

Furthermore, the reproducibility of CT number was validated over “short” (≤ 24 h) and “long” (~ 1 month) time periods, which were every two hours from 9 a.m. to 5 p.m., or once a week, respectively. Each scan was performed once.

**Table 2:** Scan parameters

*Mode*	*Scan*	*kVp*	*mA*	*Rotation Time (s)*	*SFOV*	*Slice Thickness (mm)*	*Beam Collimation (mm)*
Regular	Helical	120	630	0.5	Large body	2.5	40
GSI	Helical	80/140	600	1.0	Large body	2.5	40

SFOV = scan field of view.

**Table 3:** Reconstruction parameters of the two imaging modes

*Mode*	*DFOV (cm)*	*Recon Kernel*	*Energy*
Regular	50	Standard	N/A
GSI	50	Standard	Mono 60–140 keV

DFOV = display field of view, N/A = not applicable.

### C. Data analysis

The region of interest (ROI) measurement for each phantom rod was delineated using ImageJ software.^(17)^ Images at the center slice were analyzed. The ROI diameter was approximately 1.9 cm and its size was slightly smaller than that of the rod. Solid water‐rod CT numbers are represented by the average for the four rods in the phantom.

We first plotted the measured CT number for each material against the monochromatic imaging energy. Second, the reproducibility of the CT data for each material and monochromatic imaging energy was evaluated by using their standard deviation at short and long time periods, as indicated above. Two‐sided 68% confidence intervals were used to assess precision. Third, we plotted relative ED as a function of CT number.

The true CT numbers of the rods inserted in the phantom at each energy were computed from the mass attenuation coefficients using NIST XCOM computer program,^(^ ^18^ ^,^ ^19^ ^)^ and the mass densities are shown in Table 1. The material composition of the inserts used in the program is provided by the manufacture. The program uses the following equation:

(10) $$CT\#{\left(E_{0}\right)}_{j}=\frac{\rho_{j}\cdot \mu^{M}{\left(E_{0}\right)}_{j}-\rho_{w}\cdot \mu^{M}{\left(E_{0}\right)}_{w}}{\rho_{w}\cdot \mu^{M}{\left(E_{0}\right)}_{w}}\cdot 1000$$

where $\rho_{j}$ and $\mu^{M}{\left(E_{0}\right)}_{j}$ are the mass density of material *j* and mass attenuation coefficient of the material at energy $E_{0}$, respectively. At room temperature, $0.99823\;g/{\text{cm}}^{3}$ at 20°C was used as water mass density.

## III. RESULTS

A. Differences in CT number with different combinations of materials and monochromatic imaging energies

**Figure 2:**
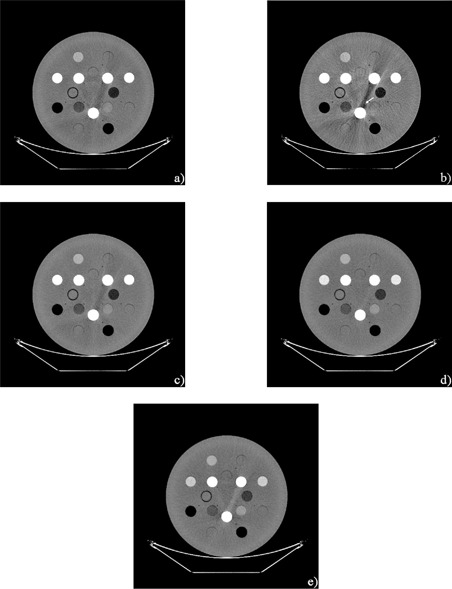
Overview of polychromatic and monochromatic images: a) 120 kVp, b) 60 keV, c) 77 keV, d) 100keV, e) 140 keV The arrow indicates an artifact appearing as a dark band around the cortical bone rod.

Figure 2 presents the CT images acquired by scanning in both modes. The 60 keV image contains an artifact appearing as a dark band around the cortical bone rod (Fig. 2(b), arrow). The magnitude of this artifact was reduced as monochromatic image energy increased. However, a slight artifact around the rod was observed at 140 keV. The image at 100 keV was visibly better in quality in this study.

**Figure 3:**
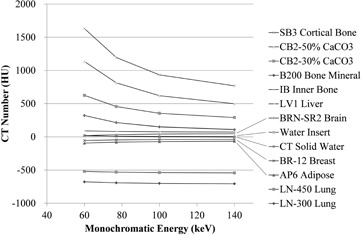
Distribution of CT number among materials as a function of monochromatic energy.

The CT number changed dramatically for high‐density material rods as shown by the monochromatic images in Fig. 3. However, the CT number varied little in the materials with densities less than or equal to water, and CT number differences for high‐energy images were less than those of lower energy images for the various material regions. The CT number range at 140 keV was about 1470 HU compared with 2300 HU at 60 keV, and was approximately 1.5‐fold smaller at high energies.

### B. CT number reproducibility over a short time period

**Figure 4:**
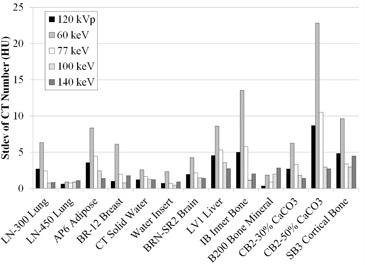
Standard deviations of CT numbers over a short time period.

Figure 4 shows standard deviations of the CT numbers over a short time period. The standard deviation at 120 kVp was close to that at 77 keV (−7.48 ± −1.56 HU, the difference averaged for all materials) and was also small at high energies: 140 keV, CB2‐50% = 2.7 HU. In contrast, this number was greater at low energies: 60 keV, CB2‐50% = 22.8 HU.

### C. CT number reproducibility over a long time period

**Figure 5:**
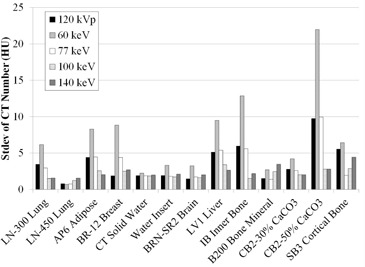
Standard deviations of CT numbers over a long time period.

Figure 5 shows standard deviations for CT numbers over a long time period. The trend was similar to that for a short time period, as stated above.

### D. CT number differences between monochromatic image and theoretical value

**Figure 6:**
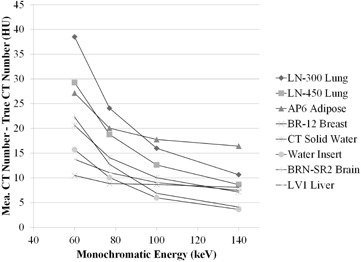
CT number difference between monochromatic image (measured) and theoretical (true) value in soft tissues.

**Figure 7:**
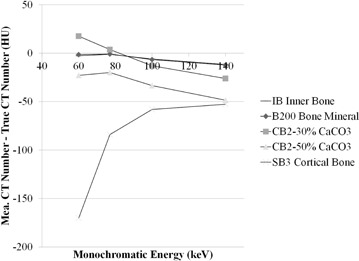
CT number difference between monochromatic image (measured) and theoretical (true) value in bony materials.

The CT number accuracy of the inserts was investigated. Figure 6 shows CT number difference between monochromatic image and theoretical (true) value in soft tissues. As the energy increases, the difference is smaller. The 140 keV monochromatic image had the least amount of CT number deviation among the materials. Figure 7 shows differences between monochromatic image and theoretical (true) CT number values in bony materials. The difference is less in images at higher energy. In SB3 cortical bone, the CT number is dramatically affected by the energy increases.

### E. CT number to ED conversion curves for monochromatic images

**Figure 8:**
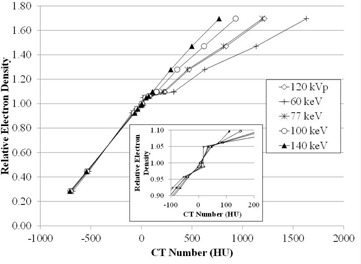
CT to ED conversion curves obtained by monochromatic images. The cutout shows the plots around 0 HU.

Figure 8 shows the CT to ED conversion curves for various monochromatic images. These curves were generated from the short time period data. The curves plotted from the data for long time periods exhibited the similar trend and are, therefore, not presented here. The standard CT image curve displayed a bilinear relationship clustering around 0 HU (Fig. 8). There were no significant differences between the curves at numbers < water (0 HU). The curves' shapes at 77 keV and 120 kVp were similar. The bilinear relationship gradually diminished with increasing energy. The curve for 140 keV was nearly linear.

## IV. DISCUSSION

Dual‐energy CT provides clinically useful, material‐specific information^(9)^ in addition to the morphological information.^(^ ^20^ ^,^ ^21^ ^)^ As a first step on the application of dual‐energy CT for RTP we evaluated the CT numbers of ED‐known materials on monochromatic images obtained by the GSI operation mode.

In the GSI mode, the “monochromatic image” is reconstructed from “monochromatic projections”, which are calculated from 80 kVp and 140 kVp projections. In other words, the monochromatic image is not obtained with monochromatic X‐ray projections, but “synthesized” with polychromatic 80 kVp and 140 kVp X‐ray projections. It should be noted that the lowest energy (e.g., 60 keV) monochromatic image is predominantly derived from 80 kVp projections than 140 kVp ones. It is well known that lower energy X‐rays are attenuated to a greater extent than higher energy X‐rays when a polychromatic X‐ray beam, especially one generated with a low tube voltage, passes through an object being imaged. This so‐called beam hardening phenomenon^(22)^ induces dark (or white) band artifacts because X‐rays from some projection angles are hardened to a differing extent than rays from other angles. This confuses the reconstruction algorithm. The most common example occurs between the dense materials, and is demonstrated in Fig. 2(a) from 120 kVp imaging. This phenomenon could be theoretically suppressed with a “true” monochromatic X‐ray, but remains in the “synthesized” monochromatic image, especially at a lower energy (Fig. 2(b)). Goodsitt et al.^(23)^ also reported that the synthesized monochromatic images are not truly monochromatic, especially at lower energy. This is partly due to the scattering X‐rays from outside the focal spot or surrounding material. The theory is not able to take into account the scatter effect while addressing the beam hardening effect. The scatter effect on dual‐energy CT was studied by Vetter and Holden.^(24)^ They found about 2% variations under different measurement conditions, and demonstrated nonlinearities in lookup tables due to the scatter effect. Their scanner had a collimator of 10 mm width. However, the GE scanner in this experiment used a 20 to 40 mm width. As a result, the scanner suffers from the scatter effect due to higher scatter fractions. In addition, the scatter fraction changes with each energy.^(24)^ Implementing better scatter correction algorithms,^(25)^ as well as increasing spectral separation between the low‐ and high‐energy X‐ray,^(^ ^4^ ^,^ ^26^ ^,^ ^27^ ^)^ could improve the synthesized monochromatic image.

To our knowledge, there have been few reports using the GE scanner to study the CT number of various materials except for water.^(^ ^23^ ^,^ ^28^ ^)^ The CT number changed dramatically for high‐density materials in the different monochromatic images (Fig. 3). This result is consistent with data acquired using iodine solutions.^(28)^ The high degree of change in CT numbers is caused by the dominance of the photoelectric effect over that of the Compton effect. The photoelectric effect probability at low energy is proportional to the atomic number cubed. In contrast, the Compton effect becomes dominant as the photon energy increases and is independent of atomic number. Therefore, the CT number varies little among diverse types of materials at high energy and explains why the CT number range is small for high‐energy images.

CT number reproducibility during short and long time periods is quite important for any application using the CT number, such as the RTP system. To our knowledge, there are no reports of the stability over a day or a month. The stability of CT number of any materials is investigated only over the short term.^(^ ^23^ ^,^ ^28^ ^)^ The trend of the CT number reproducibility was the same over a short or long time period, thus confirming the scanner stability. However, the standard deviation was somewhat large for CB2–50% at 60 keV. We consider that this might be due to the streak artifact reported by Papanikolaou et al.^(29)^ This artifact would be, as described above, due to the fact that the image is not truly monochromatic. The artifact extended in a direction toward the CB2–50% rod (Fig. 2(b)). The artifact was reduced as the energy used to generate the monochromatic image was increased. This would explain the stability of CT number at the CB2–50% rod (Figs. 4 and 5). Although the large standard deviation may also result from noise on the 60 keV image, the noise is comparatively lower, as reported by Zhang et al.,^(28)^ and would not primarily affect the standard deviation.

Image noise for given dose is also an important property. The noise determines the lower limit of subject contrast that can be distinguished by the observer. Less noise image would have more benefit for contouring objects, as well as dose calculation, in RTP Theory predicts that there is an optimal energy for which the noise in the monochromatic energy has the same energy as in a regular CT, for the same given dose.^(13)^ A study using a water‐equivalent uniform phantom shows that the monochromatic images at the optimal energy have higher noise level than diagnostic X‐ray energy range under the same acquisition and reconstruction conditions.^(28)^ In our experiment (data not shown), in the monochromatic image the standard deviation within the ROI of the water insert showed a similar trend to previous studies — the noise dramatically decreases to the optimal energy and gradually increases as the energy increases.^(^ ^13^ ^,^ ^28^ ^)^ The other inserts also showed a similar variation as the water insert. The noise of all inserts was also consistent over both short and long time periods. The noise is a very sensitive parameter to the overall imaging performance of the scanner. The scanner stability was also confirmed from this point of view.

An accurate dose calculation algorithm, as well as accurate determination of the relationship between CT number and ED, is required to accurately calculate dose distribution, minimizing discrepancy between calculated and actual dose. Venselaar et al.^(30)^ reported tolerances for the accuracy of RTP dose calculations. The accuracies required for dose calculations for homogeneous and heterogeneous media are 2% and 3%, respectively.^(30)^ The 20 HU change in the CT number for soft tissues and 250 HU for bone result in about 1% of change for monitor unit (MU) for a brain case and 2% change for a lung and pelvis case.^(29)^ The results could be transferred into the dose calculation in monochromatic image because the CT number is normalized to water. In our experiment, the results show such changes in monochromatic images at lower energy (e.g., 60 keV) between measured and true value in several tissues, but not in bony materials (Figs. 6 and 7). CT number stability results also compensate the accuracy. The study, however, shows larger difference below 60 keV in soft tissue and bone than the criteria described above (i.e., 20 HU for soft tissue and 250 HU for bone).^(23)^ That implies that the dose calculation on monochromatic images lower than 60 keV would result in larger dosimetric differences compared with other energies. The other important factor for the dose calculation is the phantom (body) size because size changes the X‐ray attenuation, as well as the amount of scatter from surrounding material, resulting in CT number change. The size has a high impact on CT number.^(31)^ Bone CT numbers under several composition variations considerably differ between small and large phantom size on monochromatic image at each energy.^(23)^ A phantom of proper size, which is close to a subsequently irradiated part such as head or body, should be scanned.

The CT to ED conversion curves with monochromatic images were determined for RTP. A linear relationship was observed at 140 keV, while the curves in the low‐energy image were bilinear, bordering around 0 HU (Fig. 8). At high energies, the Compton effect is dominant over the photoelectric effect, similar to that of megavoltage cone beam CT (MVCBCT).^(32)^ However, the dose calculation for MVCBCT has proven not to be practical because of problems, such as cupping artifacts.^(33)^ Reflecting the Compton effect compared with standard CT images used clinically for CT simulation would predict that dose calculation is performed more accurately using high‐energy monochromatic images without the problems associated with MVCBCT.^(33)^ Dosimetric investigation is required for the further evaluation of the dose calculation accuracy with these CT to ED conversion curves.

## V. CONCLUSIONS

This is the first report regarding the CT to ED conversion curves for RTP by a CT scanner with a fast kVp switching system. We present here CT to ED conversion curves acquired from monochromatic images for RTP, assessing CT number accuracy. Reproducibility was confirmed by determining the variation in CT number.

## ACKNOWLEDGMENTS

This work was supported by a Grant‐in‐Aid for Scientific Research (no. 21611004) from the Ministry of Education, Culture, Sports, Science and Technology, Japan grants.

## References

[1] Aird E , Conway J . CT simulation for radiotherapy treatment planning. Br J Radiol. 2002;75(900):937–49. 12515702 10.1259/bjr.75.900.750937

[2] Hounsfield GN . Computerized transverse axial scanning (tomography): Part 1. Description of system. Br J Radiol. 1973;46(552):1016–22. 4757352 10.1259/0007-1285-46-552-1016

[3] Rutherford RA , Pullan BR , Isherwood I . Measurement of effective atomic number and electron density using an EMI scanner. Neuroradiology. 1976;11(1):15–21. 934468 10.1007/BF00327253

[4] Rutherford RA , Pullan BR , Isherwood I . X‐ray energies for effective atomic number determination. Neuroradiology. 1976;11(1):23–28. 934469 10.1007/BF00327254

[5] Brooks RA . A quantitative theory of the Hounsfield unit and its application to dual energy scanning. J Comput Assist Tomogr. 1977;1(4):487–93. 615229 10.1097/00004728-197710000-00016

[6] Alvarez RE and Macovski A . Energy‐selective reconstructions in X‐ray computerized tomography. Phys Med Biol. 1976;21(5):733–44. 967922 10.1088/0031-9155/21/5/002

[7] Kalender WA , Perman W , Vetter J , Klotz E . Evaluation of a prototype dual‐energy computed tomographic apparatus. I. Phantom studies. Med Phys. 1986;13(3):334–39. 3724693 10.1118/1.595958

[8] Maass C , Baer M , Kachelriess M . Image‐based dual energy CT using optimized precorrection functions: a practical new approach of material decomposition in image domain. Med Phys. 2009;36(8):3818–29. 19746815 10.1118/1.3157235

[9] Santamaria‐Panga A , Duttab S , Makrogiannisc S , et al. Automated liver lesion characterization using fast kVp switching dual energy computed tomography imaging. In: Karssemeijer N and Summers RM, editors. Medical Imaging 2010: Computer Aided Diagnosis. Proc of SPIE 2010;7624.

[10] Vetter J , Perman W , Kalender WA , Mazess R , Holden J . Evaluation of a prototype dual‐energy computed tomographic apparatus. II. Determination of vertebral bone mineral content. Med Phys. 1986;13(3):340–43. 3724694 10.1118/1.595951

[11] Montner SM , Lehr JL , Oravez WT . Quantitative evaluation of a dual energy CT system. J Comput Assist Tomogr. 1987;11(1):144–50. 3805401 10.1097/00004728-198701000-00029

[12] Lehmann L , Alvarez R , Macovski A , et al. Generalized image combinations in dual KVP digital radiography. Med Phys. 1981;8(5):659–67. 7290019 10.1118/1.595025

[13] Alvarez R , Seppi E . A comparison of noise and dose in conventional and energy selective computed tomography. Nuclear Science, IEEE Transactions on. 1979;26(2):2853–56.

[14] Storm E and Israel HI . Photon cross sections from 1 keV to 100 MeV for elements Z = 1 to Z = 100. Los Alamos, NM: Los Alamos Scientific Laboratory; 1970.

[15] Slaney M and Kak A . Principles of computerized tomographic imaging. Philadelphia, PA: SIAM; 1988.

[16] Gammex Inc. Tissue characterization phantom Gammex 467 user's guide [cited 2012 12/20]. Available from: http://www.gammex.com/n‐portfolio/productpage.asp?id=283&category=Radiation+Oncology&name=Tissue+Characterization+Phantom%2C+Gammex+467

[17] Abràmoff MD , Magalhäes PJ , Ram SJ . Image processing with ImageJ. Biophotonics International. 2004;11(7):36–42.

[18] Berger M , Hubbell J , Seltzer S , et al. XCOM: photon cross sections database, NIST standard reference database 8 (XGAM). 2005 Available from: http://www.nist.gov/pml/data/xcom/index.cfm

[19] Hubbell JH . Photon cross sections, attenuation coefficients, and energy absorption coefficients from 10 keV to 100 GeV. NSRDS‐NBS29. Washington, DC: NIST; 1969.

[20] Neville AM , Gupta RT , Miller CM , Merkle EM , Paulson EK , Boll DT . Detection of renal lesion enhancement with dual‐energy multidetector CT. Radiology. 2011;259(1):173–83. 21292866 10.1148/radiol.10101170

[21] Lv PJ , Lin XZ , Li JY , Li WX , Chen KM . Differentiation of small hepatic hemangioma from small hepatocellular carcinoma: recently introduced spectral CT method. Radiology. 2011;259(3):720–29. 21357524 10.1148/radiol.11101425

[22] Bushberg JT , Seibert JA , Leidholdt EM , Boone JM . The essential physics of medical imaging. Philadelphia, PA: Lippincott Williams & Wilkins; 2001.

[23] Goodsitt MM , Christodoulou EG , Larson SC . Accuracies of the synthesized monochromatic CT numbers and effective atomic numbers obtained with a rapid kVp switching dual energy CT scanner. Med Phys. 2011;38(4):2222–32. 21626956 10.1118/1.3567509

[24] Vetter J and Holden J . Correction for scattered radiation and other background signals in dual‐energy computed tomography material thickness measurements. Med Phys. 1988;15(5):726–31. 3185409 10.1118/1.596187

[25] Cardinal HN and Fenster A . An accurate method for direct dual‐energy calibration and decomposition. Med Phys. 1990;17(3):327–41. 2385190 10.1118/1.596512

[26] Primak A , Ramirez Giraldo JC , Liu X , Yu L , McCollough C . Improved dual‐energy material discrimination for dual‐source CT by means of additional spectral filtration. Med Phys. 2009;36(4):1359–69. 19472643 10.1118/1.3083567 PMC2719491

[27] Yang M , Virshup G , Clayton J , Zhu X , Mohan R , Dong L . Does kV‐MV dual‐energy computed tomography have an advantage in determining proton stopping power ratios in patients? Phys Med Biol. 2011;56(14):4499–515. 21719949 10.1088/0031-9155/56/14/017 PMC3144258

[28] Zhang D , Li XH , Liu B . Objective characterization of GE Discovery CT750 HD scanner: gemstone spectral imaging mode. Med Phys. 2011;38(3):1178–88. 21520830 10.1118/1.3551999

[29] Papanikolaou N , Battista J , Boyer A , et al. Tissue inhomogeneity corrections for megavoltage photon beams. AAPM Task Group 65. Madison (WI): Medical Physics Publishing; 2004.

[30] Venselaar J , Welleweerd H , Mijnheer B . Tolerances for the accuracy of photon beam dose calculations of treatment planning systems. Radiother Oncol. 2001;60(2):191–201. 11439214 10.1016/s0167-8140(01)00377-2

[31] Joseph PM and Spital RD . The effects of scatter in x‐ray computed tomography. Med Phys. 1982;9(4):464–72. 7110075 10.1118/1.595111

[32] Petit SF , van Elmpt WJC , Nijsten SM , Lambin P , Dekker AL . Calibration of megavoltage cone‐beam CT for radiotherapy dose calculations: correction of cupping artifacts and conversion of CT numbers to electron density. Med Phys. 2008;35(3):849–65. 18404922 10.1118/1.2836945

[33] Morin O , Chen J , Aubin M , et al. Dose calculation using megavoltage cone‐beam CT. Int J Radiat Oncol Biol Phys. 2007;67(4):1201–10. 17336221 10.1016/j.ijrobp.2006.10.048

